# Bisphenol A induces ototoxic injury by ferroptosis pathway: integrated analysis of network toxicology, RNA-sequencing and experimental validation

**DOI:** 10.3389/fnmol.2026.1829676

**Published:** 2026-07-28

**Authors:** Juanjuan Li, Dunhui Yang, Suwen Bai, Xianhai Zeng, Peng Zhang

**Affiliations:** Department of Otolaryngology, Shenzhen Longgang Otolaryngology Hospital, Shenzhen Otolaryngology Research Institute, Shenzhen, Guangdong, China

**Keywords:** Bisphenol A, environmental pollutant, ferroptosis, network toxicology, ototoxic injury

## Abstract

Bisphenol A, a globally utilized plastic monomer, has extensively infiltrated aquatic and atmospheric environments, emerging as a high-priority environmental pollutant. However, its toxicological impact on ototoxic injury and the associated molecular mechanisms remain largely unexplored. In this study, we employed an integrative approach combining network toxicology, RNA sequencing, and cellular phenotyping to systematically elucidate the pathways of Bisphenol A-induced injury. Potential ototoxicity-related targets and Bisphenol A-binding proteins were sourced from databases such as BindingDB, CTD, SwissTargetPrediction, TargetNet, DisGeNET, and GeneCards, resulting in the identification of 55 candidate targets. Functional enrichment analysis revealed significant involvement in autophagy and HIF-1 signaling pathways, while multi-algorithm topology analysis identified CASP3, PTGS2, CYCS, BCL2, TNF, PPARG, NFE2L2, and MAPK3 as key hub proteins. Experimental validation demonstrated that Bisphenol A reduced cell viability and increased lactate dehydrogenase leakage in a concentration-dependent manner. RNA sequencing further revealed a marked activation of the ferroptosis pathway following Bisphenol A exposure. Ferroptotic cell death was further corroborated by increased levels of lipid reactive oxygen species, malondialdehyde, and intracellular Fe^2+^, accompanied by the down-regulation of GPX4, SLC3A2, and FSP-1 expression, as well as the up-regulation of ACSL4, PTGS2, and HO-1 expression. Collectively, our findings indicate that Bisphenol A induces ototoxic injury via the ferroptosis pathway, suggesting that this environmental contaminant may contribute to hearing loss and highlighting potential interventions against Bisphenol A exposure.

## Introduction

Ototoxicity refers to damage to the inner ear caused by certain pharmaceuticals or environmental pollutants, resulting in temporary or permanent hearing loss, tinnitus, and vestibular dysfunction (Steyger, 2021). Previous research has predominantly focused on pharmaceutical ototoxicants, such as aminoglycoside antibiotics, platinum-based chemotherapeutics, and salicylates, which are now recognized as the primary iatrogenic contributors to hearing impairment (Kim et al., 2022; Müller et al., 2003; Tan and Vlajkovic, 2023). The underlying mechanisms of these agents involve mitochondrial dysfunction and oxidative stress. For instance, aminoglycosides induce an overload of Ca^2+^ in cochlear hair cells, triggering Caspase-dependent apoptotic pathways, whereas cisplatin significantly increases intracellular reactive oxygen species (ROS) through the NADPH oxidase system, leading to cellular damage (Chen L. et al., 2024; Kim et al., 2010). The rapid pace of industrialization has considerably increased the exposure to heavy metals, organic solvents, and microplastics, shifting the focus toward environmentally induced ototoxicity (Rosati and Jamesdaniel, 2020). Epidemiological studies reveal that a substantial proportion of exposures to lead (75%), styrene (74%), and toluene (77%) significantly heighten the risk of auditory damage (Hemmativaghef, 2020). Chronic lead exposure raises auditory brainstem response thresholds, provokes hair-cell malformation, and intensifies oxidative stress (Hu et al., 2019). Styrene exhibits a gradient distribution along the cochlea, activates apoptotic signaling pathways, and causes a dose-dependent loss of outer hair cells in the mid-frequency region, ultimately resulting in permanent hearing loss (Sullivan et al., 1988). Of growing concern, microplastics, now pervasive in the environment, have not been thoroughly investigated for their ototoxic potential; their underlying mechanisms remain largely unexplored and necessitate systematic investigation.

Bisphenol A (2,2-bis(4-hydroxyphenyl)propane, C_15_H_16_O_2_, MW:228.29), a lipophilic small-molecule plastic monomer widely used in global manufacturing, enters the human body through ingestion, inhalation, and dermal absorption, leading to lifelong low-dose cumulative exposure (Abraham and Chakraborty, 2020). Epidemiological and experimental studies have linked Bisphenol A to various pathologies: it exacerbates psoriasis through immune system interference, impairs infant neurodevelopment following prenatal exposure, and promotes tumorigenesis in breast cancer via the CXCL12/AKT signaling axis (Braun, 2017; Dong et al., 2025; Feng et al., 2025). In the field of otology, only cross-sectional studies have associated childhood Bisphenol A exposure with reduced cochlear hair-cell activity (Chang et al., 2024); whether Bisphenol A can directly damage these cells, as well as the key molecular targets and regulatory networks involved, remains entirely unexplored and demands systematic investigation.

In contrast to the traditional “single-compound-single-target” model, which faces challenges in elucidating the complex multi-pathway toxicity of environmental pollutants, network toxicology leverages multi-omics data to construct comprehensive interaction networks that connect chemical exposure, toxic phenotypes, and molecular targets. This approach has been extensively utilized in research on inflammation, cancer, and other diseases (Li et al., 2025). In the present study, we applied the network toxicology framework to investigate Bisphenol A on ototoxic injury. We systematically integrated ototoxicity-related targets and potential Bisphenol A targets from databases such as BindingDB, CTD, SwissTargetPrediction, TargetNet, and GeneCards. Subsequently, we constructed a “Bisphenol A-ototoxicity” pathogenic network and identified core targets using multiple algorithms, including MNC, MCC, and Degree. We validated the cytotoxic effects of Bisphenol A in a cochlear hair cell model and explored mechanistic pathways via RNA sequencing, thereby achieving a comprehensive cycle of “network prediction, transcriptomic discovery, and cellular validation.”

## Materials and methods

### Collect potential targets of Bisphenol A

We downloaded the chemical structure, 2D and 3D representations of Bisphenol A from PubChem^1^ and subsequently predicted and collected its putative targets using BindingDB^2^, CTD^3^, SwissTargetPrediction^4^, and TargetNet^5^ (He et al., 2024; Kim, 2016).

### Acquire potential ototoxicity-related targets

Using “Ototoxicity” as the search term, we systematically retrieved and integrated potential ototoxicity-related targets from the DisGeNET and GeneCards databases (Liao et al., 2022). Identify potential targets underlying Bisphenol A-induced ototoxicity. By integrating the potential targets of Bisphenol A with ototoxicity-related genes, we used the “VennDiagram” R package to extract their intersection as compound–disease targets. These shared targets were then uploaded to the STRING database^6^ to construct a protein–protein interaction (PPI) network (Szklarczyk et al., 2023).

### Bisphenol A toxicity prediction

The SMILES string of Bisphenol A was submitted to the ProTox 3.0 webserver^7^ for *in silico* calculation, yielding systematic predictions of its toxicological profile and susceptible organs (Banerjee et al., 2024).

### Functional pathway analysis of Bisphenol A-induced ototoxicity targets

To explore the potential functional pathways involved in Bisphenol A-induced ototoxicity, we performed Gene Ontology (GO) and Kyoto Encyclopedia of Genes and Genomes (KEGG) enrichment analyses using the clusterProfiler R package. Pathways with q < 0.05 were considered statistically significant and retained for further interpretation.

### Identification of potential core targets for Bisphenol A-induced ototoxicity using multiple algorithms

The potential targets associated with Bisphenol A-induced ototoxicity were imported into Cytoscape, and the MCODE plug-in was used to extract the most significant sub-network under the following parameters: degree cutoff = 2, node score cutoff = 0.2, k-core = 2, and max. depth = 100. Subsequently, the CytoHubba plug-in was employed to rank nodes by Maximum Neighborhood Component (MNC), Maximum Clique Centrality (MCC), and Degree centrality (Degree). The core targets identified by each algorithm were intersected to generate the final set of putative hub genes for Bisphenol A-induced ototoxicity.

### Cell culture

Mouse cochlear hair-cell line HEI-OC1 was obtained from BNCC. Cells were routinely maintained in DMEM supplemented with 10 % fetal bovine serum, 100 U ml^–1^ penicillin, and 100 μg ml^–1^ streptomycin at 37 °C in a humidified incubator containing 5 % CO_2_.

### Experimental reagents

Bisphenol A (B108652; 2,2-bis(4-hydroxyphenyl)propane) was purchased from Aladdin™. The compound was dissolved in DMSO (Solarbio) to prepare a 200 mM stock solution. Immediately before use, aliquots were diluted to the desired working concentrations in complete medium; the final DMSO concentration never exceeded 0.1%, a level shown to be non-cytotoxic. Primary antibodies against ACSL4 (22401-1-AP), SLC3A2 (15193-1-AP), PTGS2 (27308-1-AP), HO-1 (10701-1-AP), FSP-1 (20886-1-AP), GPX4 (67763-1-Ig) were obtained from Proteintech; anti-β-actin (sc-47778) was from Santa Cruz Biotechnology. Liproxstatin-1 (HY-12726), Ferrostatin-1 (HY-100579) and Z-VAD-FMK (HY-16658B) were purchased from MedChemExpress.

### Cell viability assay

CCK-8 kit (MedChemExpress) was used according to the manufacturer’s instructions. After exposure to the indicated treatments for 24 or 48 h, 10 μL of CCK-8 reagent was added to each well and the plates were incubated at 37 °C for 2 h. Absorbance was then measured at 450 nm using a microplate reader.

### Colony formation assay

Cells were trypsinized, counted, and seeded at 800 cells per well in six-well plates. After attachment, they were exposed to the indicated treatments and cultured for 2 weeks. Colonies were fixed with 4% paraformaldehyde (Biosharp) for 15 min, and then stained with 1% crystal-violet solution (Beyotime Biotech) for 20 min, rinsed thoroughly with water, air-dried, and counted using ImageJ.

### LDH cytotoxicity assay

To evaluate Bisphenol A-induced cytotoxicity, we used the Lactate Dehydrogenase (LDH) Cytotoxicity Assay Kit (Beyotime Biotech). Briefly, after exposing to Bisphenol A. LDH release was then quantified according to the manufacturer’s instructions.

### Western blotting

Western blotting was performed as previously described (Zeng et al., 2024). Treated cells were lysed with RIPA buffer (Sericebio) to extract total protein, and the protein concentration was determined using a BCA assay kit (Biosharp).

### RNA-seq

Total RNA from treated cells was quantified and assessed for integrity with an Agilent 2100 Bioanalyzer using the Agilent RNA 6000 Nano Kit. Samples that passed quality control were subjected to transcriptome sequencing on the BGISEQ-500 platform by ID-Biotch.

### Malondialdehyde (MDA) and lipid reactive oxygen species assays

In this study, intracellular malondialdehyde (MDA) and lipid reactive oxygen species levels were measured using the MDA Assay Kit (S0131S, Beyotime Biotech) and the C11-BODIPY 581/591 probe (D3861, Thermo Fisher Scientific), respectively.

### Fe^2+^ detection

After gentle washing with PBS, cells were incubated with FerroOrange dye (F374, Dojindo) according to the manufacturer’s instructions for 30 min at 37 °C in a cell incubator. Images were acquired using a confocal microscope, and the mean fluorescence intensity was quantified with ImageJ to reflect intracellular free Fe^2+^ levels.

### Statistical analysis

Statistical analyses were performed with R (v4.3.2; R Foundation for Statistical Computing, Vienna, Austria) and GraphPad Prism (v10.1.2; GraphPad Software Inc.). All experiments were carried out in at least three independent replicates. Data are presented as mean ± SEM. Comparisons between two groups were evaluated by Student’s *t*-test, and comparisons among three or more groups were analyzed by one-way ANOVA. *P* < 0.05 was considered statistically significant.

## Results

### Acquisition of potential targets for Bisphenol A and ototoxicity and toxicity prediction

**FIGURE 1:**
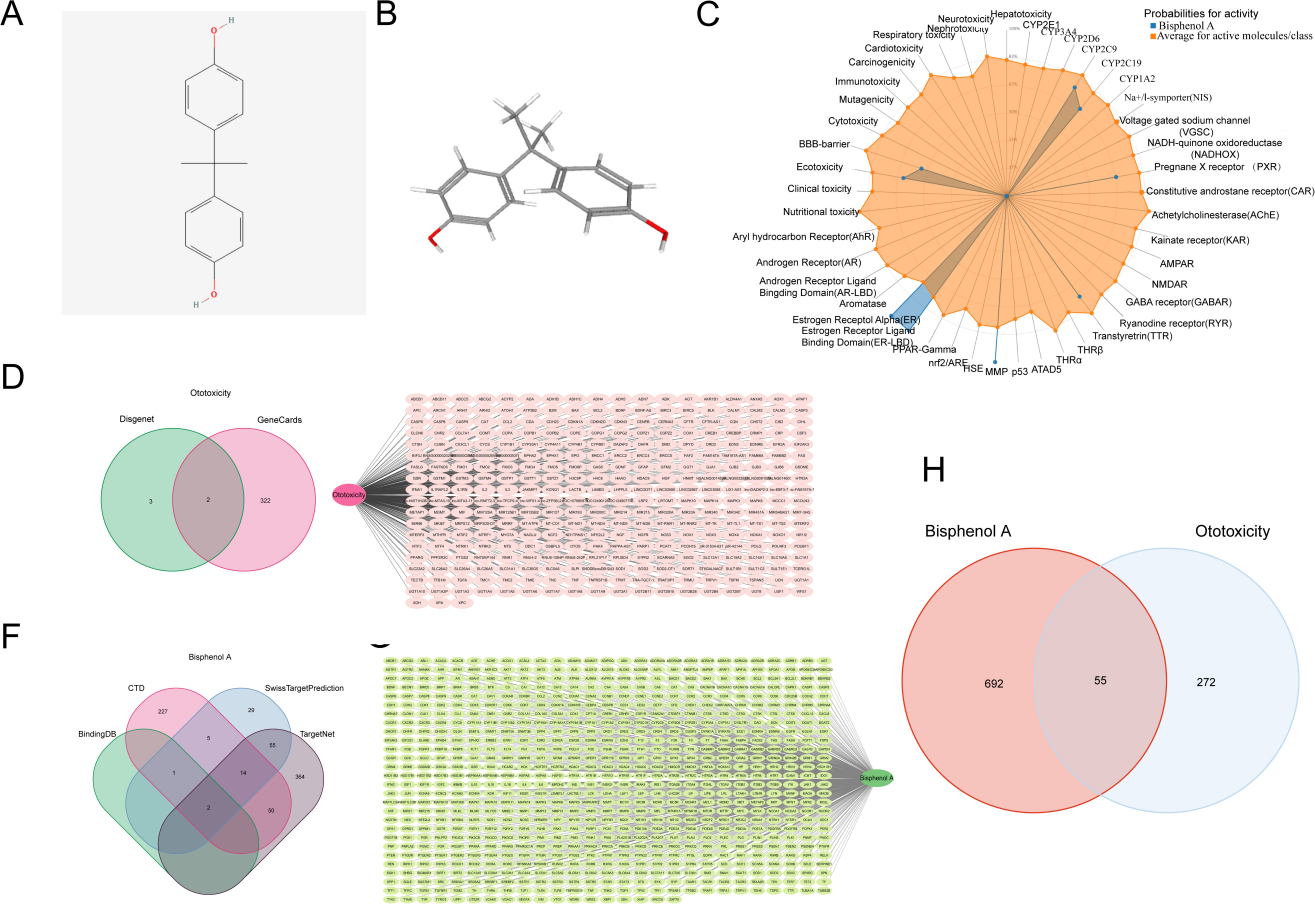
Chemical structures and toxicological prediction of Bisphenol A. **(A)** 2-D structure of Bisphenol A. **(B)** 3-D structure of Bisphenol A. **(C)** Toxicological prediction profile of Bisphenol A. **(D,E)** Collection of 327 ototoxicity-related targets from DisGeNET and GeneCards databases. **(F,G)** Collection of 747 Bisphenol A-related targets from BindingDB, CTD, SwissTargetPrediction, and TargetNet databases. **(H)** Venn diagram showing the 55 overlapping targets common to both Bisphenol A and ototoxicity datasets.

By searching “Bisphenol A” in PubChem we obtained its SMILES string (CC(C)(C1 = CC = C(C = C1)O)C2 = CC = C(C = C2)O) together with 2-D and 3-D structures (Figures 1A, B). The compound was then submitted to ProTox 3.0 for toxicity prediction (Figure 1C). High-confidence alerts were returned for estrogen receptor (ER), estrogen-receptor ligand-binding domain (ER-LBD) and MMP, indicating that Bisphenol A can modulate calcium channels via estrogen signaling and may disturb the electrochemical gradient across the inner and outer mitochondrial membranes, leading to loss of membrane potential and precipitating early oxidative stress. A medium-confidence score for blood-brain barrier (BBB) permeability suggests that Bisphenol A can cross the BBB and accumulate at low doses in the cochlea over time (Supplementary Table 1). To identify potential targets through which Bisphenol A might elicit ototoxicity, we compiled 327 ototoxicity-related genes retrieved from DisGeNET and GeneCards (Figures 1D, E) and 747 putative Bisphenol A targets predicted by BindingDB, CTD, SwissTargetPrediction and TargetNet (Figures 1F, G). Intersection analysis with the VennDiagram R package yielded 55 genes proposed as candidate targets for Bisphenol A-induced ototoxicity (Figure 1H).

### Identification of key targets and pathways in Bisphenol A-induced ototoxicity

**FIGURE 2:**
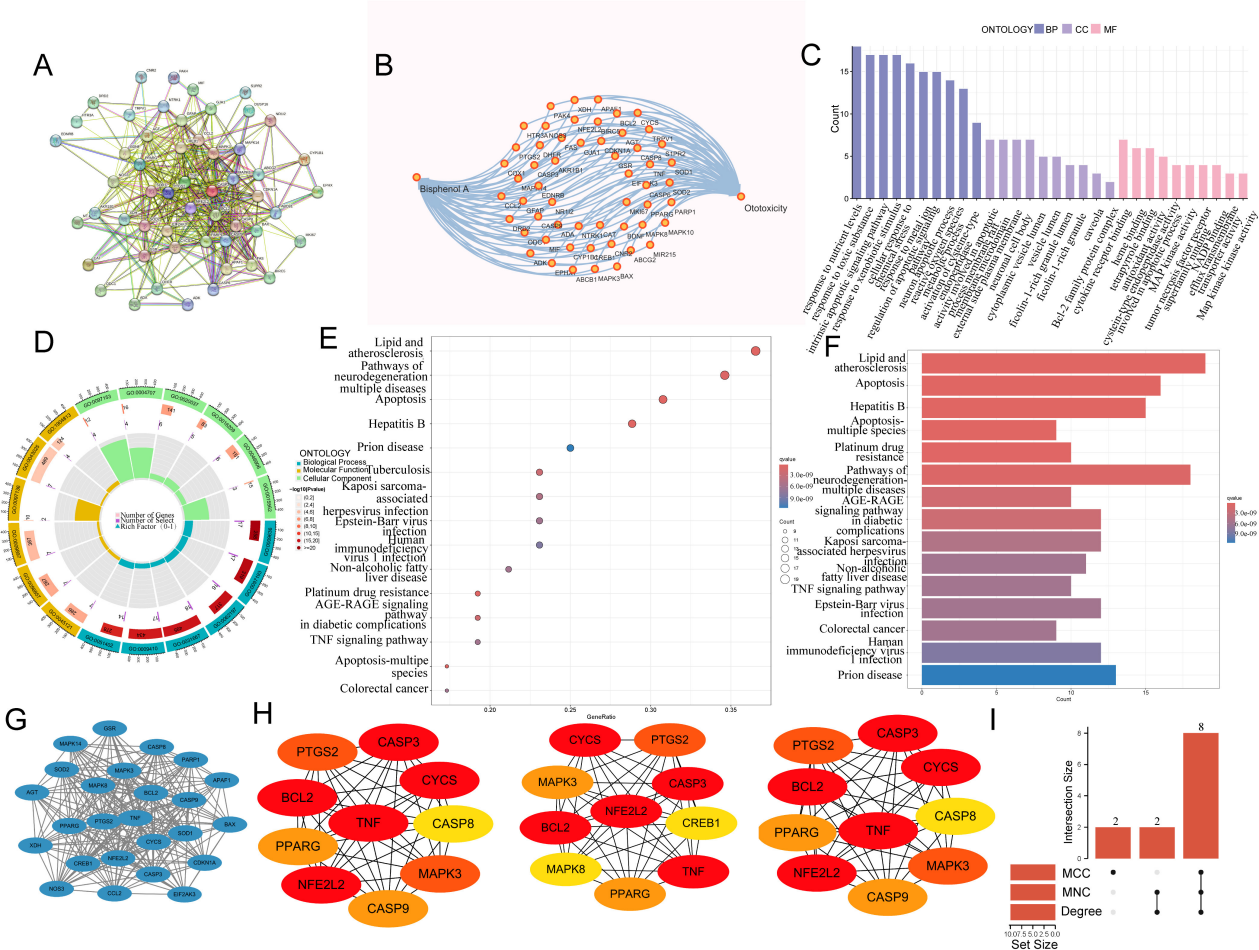
Construction of the Bisphenol A-induced ototoxicity PPI network and exploration of potential functions. **(A,B)** Protein-protein interaction network of the overlapping Bisphenol A and ototoxicity targets. **(C,D)** Significantly enriched GO terms for the overlapping targets. **(E,F)** Significantly enriched KEGG pathways for the overlapping targets. **(G)** Core sub-network extracted by MCODE. **(H)** Top 10 hub nodes within the sub-network ranked by Degree, MCC, and MNC algorithms. **(I)** Intersection of the hub lists generated by Degree, MCC, and MNC algorithms visualized with an Upset plot.

To pinpoint the key drivers of Bisphenol A-induced ototoxicity, the 55 overlapping targets were uploaded to the STRING database to construct a protein–protein interaction network (Figure 2A) and the resulting file was imported into Cytoscape for visualization and downstream analyses (Figure 2B). GO and KEGG pathway enrichment analyses were then performed with the same 55 genes to explore the biological processes involved (Figures 2C–F). GO terms were enriched for intrinsic apoptotic signaling pathway, regulation of mitochondrial membrane potential, glutathione metabolic process, and response to metal ion (Supplementary Table 2), whereas KEGG pathways were enriched for Necroptosis, HIF-1 signaling, and Pathways of neurodegeneration (Supplementary Table 3), implying that Bisphenol A may trigger hair-cell death by disrupting mitochondrial membrane potential and increasing ROS. To identify core regulatory nodes, the most significant sub-network was extracted with the MCODE plug-in, and hub genes within this module were ranked by MNC, MCC, and Degree algorithms (Figures 2G–I). CASP3, PTGS2, CYCS, BCL2, TNF, PPARG, NFE2L2, and MAPK3 emerged as the central targets of the Bisphenol A-Ototoxicity network, occupying pivotal positions in caspase, mitochondrial, antioxidant, and inflammatory signaling cascades.

### Bisphenol A suppresses HEI-OC1 cell viability *in vitro*

**FIGURE 3:**
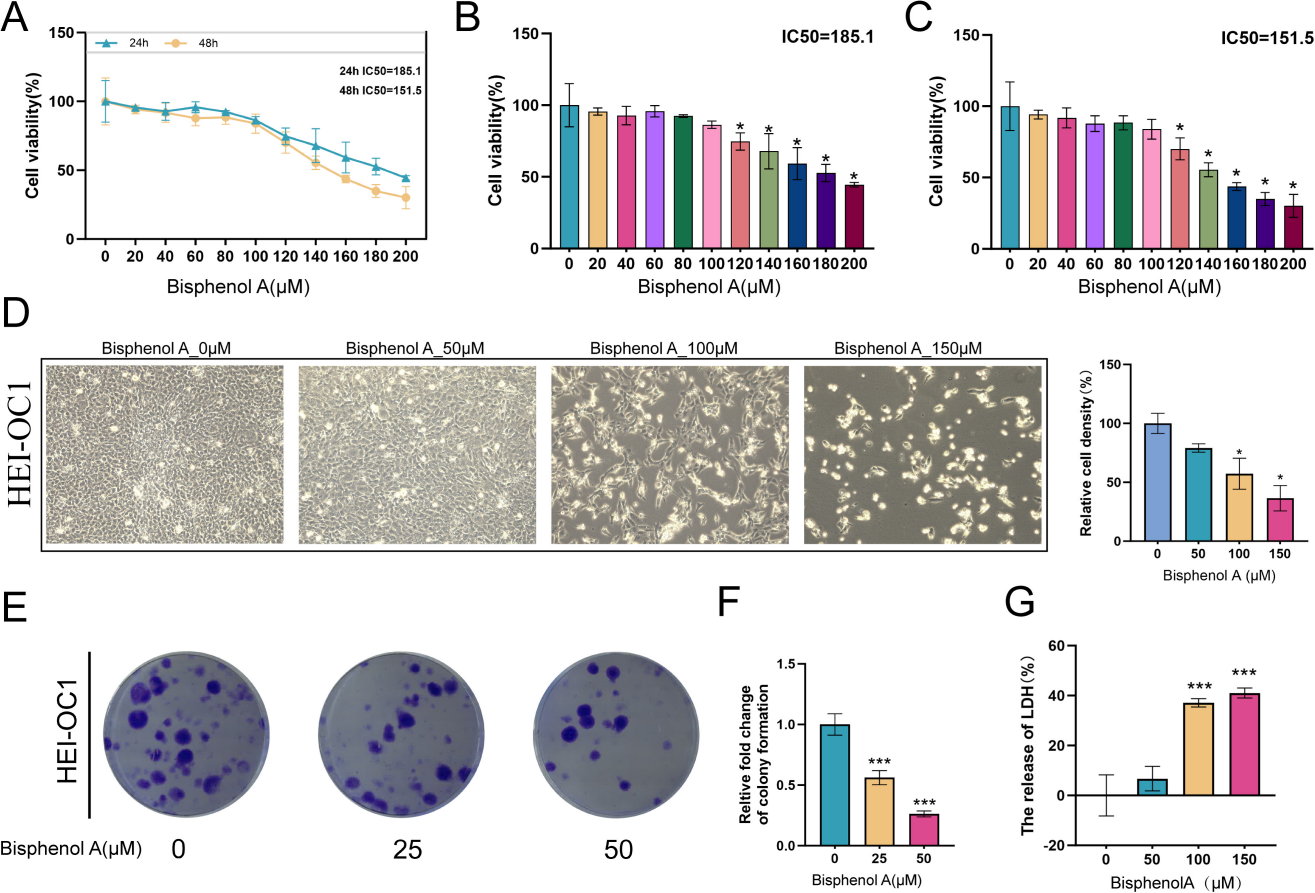
Bisphenol A suppresses HEI-OC1 proliferation *in vitro*. **(A–C)** Cell viability and IC_50_ values after Bisphenol A treatment for 24/48 h. (one-way ANOVA). **(D)** Representative micrographs quantitative analysis of HEI-OC1 treated with 0, 50, 100, or 150 μM Bisphenol A for 48 h. **(E)** Colony-formation assay: representative images and quantification. **(F)** Statistical results of colony numbers. (one-way ANOVA). **(G)** Percentage of LDH released into the culture supernatant. (one-way ANOVA) *p < 0.05 vs 0 μM, ***p < 0.001 vs 0 μM.

To validate the network-toxicology predictions, functional assays were performed in HEI-OC1 cells. CCK-8 results showed that Bisphenol A reduced cell viability in a dose-dependent manner (Figures 3A–C and Supplementary Figure 1); the 24 h IC_50_ was 185.1 μM and the 48 h IC_50_ was 151.5 μM. Light-microscopy observations revealed that cell density began to decline at 50 μM; at 100 μM the density was markedly lower and bright, refractile vacuolated cells appeared; at 150 μM cells were sparse and vacuolation was prominent (Figure 3D). Colony-forming assays confirmed that 25 and 50 μM Bisphenol A significantly decreased the number of colonies in a dose-dependent fashion (Figures 3E, F). The LDH-release assay showed that LDH activity in the culture supernatant rose significantly and dose-dependently after 48 h exposure to 50, 100, or 150 μM Bisphenol A compared with the control (Figure 3G). Collectively, Bisphenol A markedly suppresses HEI-OC1 cell viability *in vitro*.

### RNA-seq reveals that Bisphenol A activates the ferroptosis pathway

**FIGURE 4:**
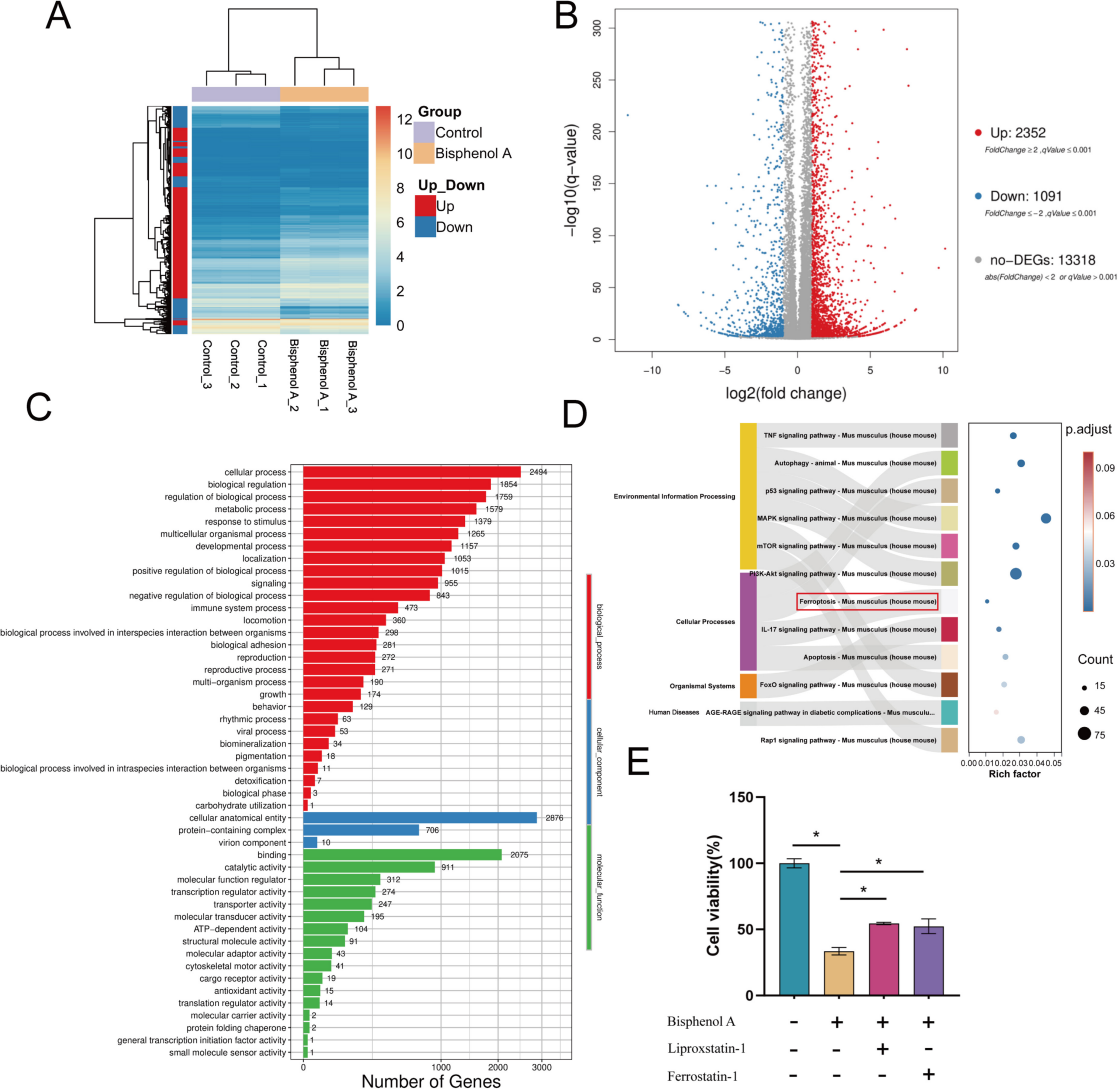
RNA-seq reveals transcriptomic changes in HEI-OC1 after Bisphenol A treatment. **(A)** Heatmap of differentially expressed genes. **(B)** Volcano plot of differentially expressed genes. **(C)** GO enrichment terms. **(D)** Sankey diagram of KEGG enrichment. **(E)** HEI-OC1 cells were pre-treated for 2 h with either Ferrostatin-1 (2 μM) or Liproxstatin-1 (2 μM), or a combination of both, followed by the addition of bisphenol A (150 μM) and further culture for 48 h. A separate inhibitor control group (without BPA) was established. Cell viability in each group was assessed using the CCK-8 assay. *p < 0.05 vs. Control (one-way ANOVA).

To uncover novel mechanisms underlying Bisphenol A-induced HEI-OC1 death, RNA-seq was performed on cells treated with 150 μM Bisphenol A for 48 h (Figures 4A, B). Differential analysis identified 3,443 differentially expressed genes (| log_2_FC| > 1, q < 0.05), comprising 2,352 up-regulated and 1,091 down-regulated genes. GO enrichment indicated significant overrepresentation of cellular process, cellular anatomical entity, and catalytic activity (Figure 4C). KEGG mapping highlighted TNF signaling, autophagy, p53 signaling, FoxO signaling, and critically ferroptosis, aligning closely with the oxidative-stress signature predicted by network toxicology. These data implicate ferroptosis as a key route through which Bisphenol A elicits ototoxicity (Figure 4D). Subsequently, the cells were pre-treated with two ferroptosis inhibitors, Ferrostatin-1 (2 μM) and Liproxstatin-1 (2 μM) for 2 h, followed by exposure to Bisphenol A (150 μM) for 48 h. The results from the CCK-8 assay indicated that both ferroptosis inhibitors partially restored cell viability (Figure 4E). These findings suggest that Bisphenol A may activate the ferroptosis pathway, leading to damage in HEI-OC1 cells, and that this pathway could represent a potential target for therapeutic intervention.

### Experimental validation of Bisphenol A-induced ferroptosis

**FIGURE 5:**
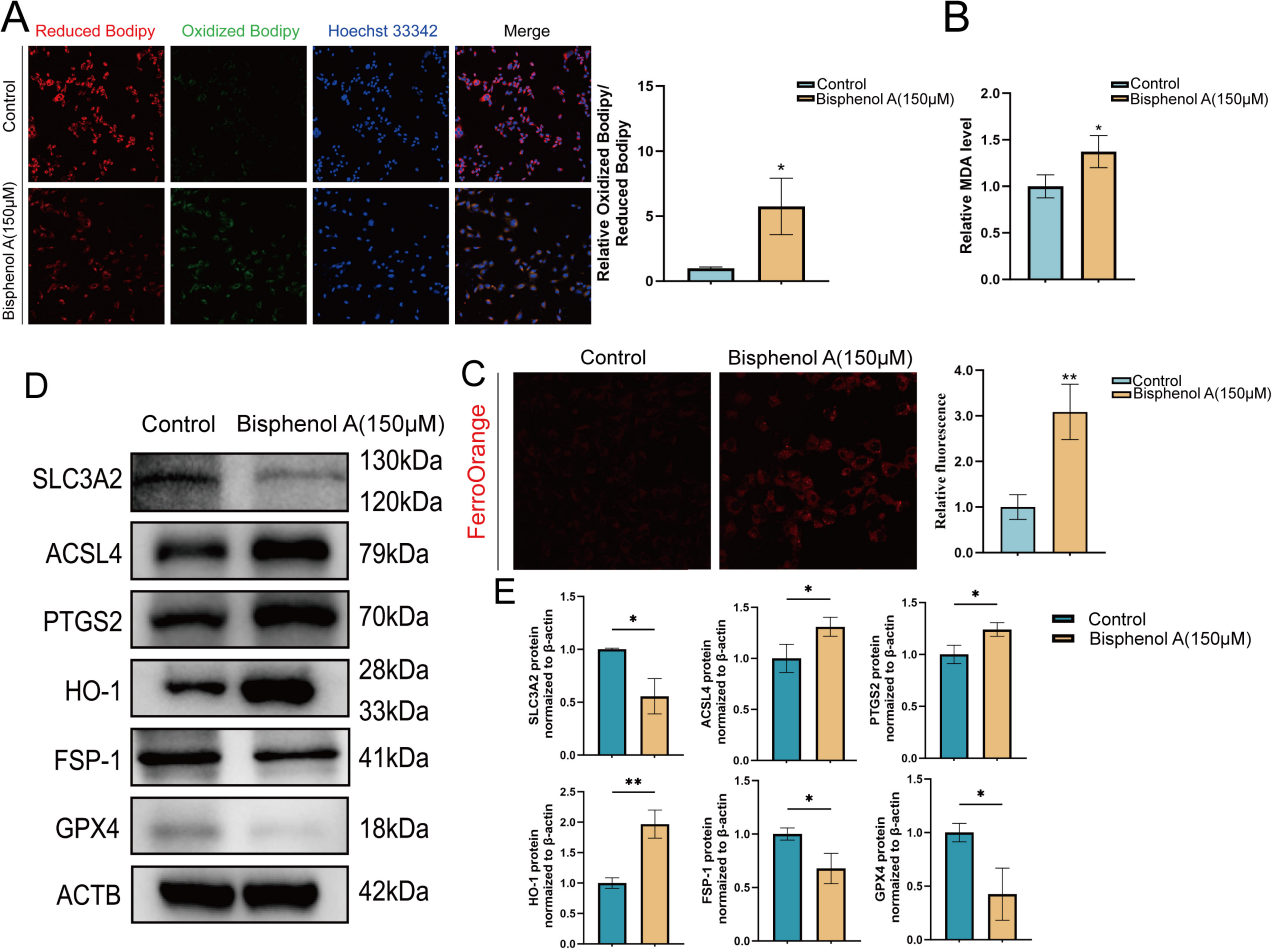
Experimental validation of Bisphenol A-induced ferroptosis in HEI-OC1 cells. **(A)** Lipid ROS detected by C11-BODIPY 581/591 probe and the summary data. (Student’s *t*-test). **(B)** MDA content measurement. (Student’s *t*-test). **(C)** FerroOrange staining for intracellular free Fe^2+^ level and the summary data. (Student’s *t*-test). **(D,E)** Representative images and western blot analysis of ferroptosis-related protein expression. (Student’s *t*-test) *p < 0.05 vs. Control, **p < 0.01 vs. Control.

To verify the RNA-seq prediction, we examined multiple ferroptosis-related indices. The lipid-oxidation probe C11-BODIPY 581/591 showed that after 48 h of 150 μM Bisphenol A treatment the reduced (red) fluorescence decreased while the oxidized (green) fluorescence markedly increased, yielding a significantly higher oxidized/reduced ratio (Figure 5A). Consistently, MDA content rose substantially in the 150 μM Bisphenol A group, confirming intensified lipid peroxidation (Figure 5B). Intracellular free Fe^2+^ was monitored with FerroOrange; fluorescence was significantly stronger in Bisphenol A-treated cells than in controls (Figure 5C), indicating elevated labile iron that fuels ferroptosis. Western blot analysis revealed a pro-ferroptotic protein profile: GPX4, SLC3A2, and FSP-1 were sharply down-regulated, whereas ACSL4, PTGS2, and HO-1 were up-regulated (Figures 5D, E). These data demonstrate that Bisphenol A cripples the antioxidant system, increases free iron, and accelerates lipid peroxidation, thereby driving HEI-OC1 cells into ferroptosis, an result that fully consistent with our bioinformatic analyses.

## Discussion

Ototoxic injury refers to structural or functional damage to the inner ear resulting from exposure to chemical agents, pharmaceuticals, or physical factors (Steyger, 2021). Given that the human inner ear is responsible for both auditory and vestibular functions, ototoxic damage typically manifests as hearing loss and tinnitus, often accompanied by vertigo and balance disturbances (Laurell, 2019). Traditionally, research has focused on the ototoxic effects of aminoglycosides, cisplatin, and noise exposure. However, the rapid pace of industrialization has significantly increased environmental levels of heavy metals, organic solvents, and microplastics, thereby drawing increased attention to their potential ototoxic risks. Bisphenol A, a globally utilized plasticizer, has become pervasive in the environment. Numerous studies have associated Bisphenol A exposure with the onset and progression of various diseases, including oxidative stress-induced male germ-cell death, increased invasiveness of papillary thyroid carcinoma, and its role as an environmental factor in Alzheimer’s disease (Zhang et al., 2026). In terms of ototoxicity, cross-sectional studies have identified a correlation between childhood exposure to Bisphenol A and hearing impairment, although the precise mechanisms of its toxic effects on the inner ear remain to be elucidated.

In this study, we initially used extensive databases to identify that Bisphenol A could interfere with calcium signaling through estrogen-like activity and directly disrupt the mitochondrial electrochemical gradient, thereby inducing early oxidative stress. Consistent with our results, Bisphenol A disrupts intracellular calcium homeostasis in human embryonic stem cell-derived cortical neurons and impairs mitochondrial function, leading to the senescence and of human amniotic mesenchymal stromal cells (Ficai et al., 2025; Wang et al., 2019). We suggest that similar processes are employed by inner-ear cells to trigger damage. Interestingly, the toxicity prediction showed medium-confidence BBB permeability (Santoro et al., 2019). Due to its high lipophilicity, Bisphenol A is capable of diffusing across membranes, traversing the BBB, entering inner-ear lymph and blood vessels, and gradually accumulating in cochlear hair cells (Hayashi et al., 2015). Concurrently, Bisphenol A exhibits low water solubility, limited renal filtration and clearance, and an extended half-life, potentially leading to chronic low-dose exposure in the ear (Chen et al., 2016). Go and KEGG enrichment analysis revealed significant associations with the intrinsic apoptotic signaling pathway, regulation of mitochondrial membrane potential, glutathione metabolic process, and response to metal ions, all of which are related to oxidative stress and mitochondrial damage. To pinpoint hub genes within the PPI network, we utilized the MCODE algorithm to extract eight core genes (CASP3, PTGS2, CYCS, BCL2, TNF, PPARG, NFE2L2, and MAPK3), which were identified as critical nodes involved in lipid oxidation, inflammation, and mitochondrial homeostasis. Based on these network toxicology insights, Bisphenol A exposure may disrupt lipid oxidation and mitochondrial homeostasis to contribute ototoxic development.

Various *in vitro* studies investigated the toxicity of Bisphenol A across different cell lines, revealing that the response sensitivity differs based on the cell type. Here, we evaluated the impact of Bisphenol A on HEI-OC1 cells and found that Bisphenol A suppressed cell growth in a dose-dependent manner, with 24 h IC_50_ = 185.1 μM and 48 h IC_50_ = 151.5 μM. In our study, we found the BPA concentrations below 100 μM did not influence cell viability, but the BPA concentrations above 120 μM suppressed cell growth. The BPA concentrations used in this study were higher than environmentally and physiologically relevant exposure levels (Cull and Winn, 2025). However, the cytotoxic concentrations used in previous studies were in line with the ranges we employed in this study. Wang et al. (2023) reported 50 μM BPA decreased growth and survival rate in *Caenorhabditis elegans* by inducing neurotransmission abnormity and oxidative damage (Wang et al., 2023). The presence of 100 μM BPA led to a reduction in dendritic and axonal fibers and modified the shape of neurons in cultures (Phoolmala et al., 2026). Moreover, HEI-OC1 cells may have reduced BPA uptake or enhanced efflux compared to other cell types, or that the short exposure duration (48 h) necessitates higher concentrations. Future study should measure intracellular BPA levels via LC-MS/MS to establish a dose-response relationship. These concentrations, while potentially higher than normal environmental exposure levels, are still important for studying the mechanisms behind acute or subacute cellular responses. Subsequently, HEI-OC1 cells exposed to Bisphenol A were subjected to RNA-seq, identifying 2,352 up-regulated and 1,091 down-regulated differentially expressed genes. GO and KEGG enrichment analysis revealed that cellular process, cellular anatomical entity, catalytic activity and TNF, p53, PI3K-Akt, and FoxO signaling pathways were key pathways; notably, the ferroptosis pathway was also significantly enriched, consistent with our network toxicology predictions (Chen J. et al., 2024). To verify whether the ferroptosis pathway indicated by bioinformatic analysis was indeed activated, we conducted a series of functional assays *in vitro*. First, C11-BODIPY 581/591 showed a significant increase in lipid ROS levels after Bisphenol A treatment, accompanied by elevated MDA content, indicating exacerbated lipid peroxidation. FerroOrange staining further confirmed a marked rise in intracellular Fe^2+^ levels, providing catalytic substrates for the Fenton reaction. At the protein level, decreased GPX4 and SLC3A2 reflected depletion of the GSH system, whereas up-regulation of ACSL4 and PTGS2 promoted peroxidation of polyunsaturated fatty-acid phospholipids, together pointing to canonical molecular features of ferroptosis (He et al., 2023; Zhang et al., 2024). Additionally, increased HO-1 and reduced FSP-1 indicated persistent iron loading and weakened antioxidant capacity, collectively demonstrating the occurrence of ferroptosis (Kong et al., 2026). However, the study is subject to several limitations. An animal model has not yet been employed to verify whether Bisphenol A exposure leads to hair-cell loss, as assessed by auditory brainstem response and distortion product otoacoustic emissions testing. Future research will incorporate in vivo models to validate this mechanism.

## Conclusion

The current findings offer a novel theoretical foundation and strategic framework for the clinical diagnosis and treatment of Bisphenol A-induced ototoxic injury. This study substantiates that Bisphenol A provokes ototoxic injury by initiating ferroptosis in cochlear hair cells, thereby inhibiting proliferation and promoting cell death. These findings not only elucidate the mechanisms underlying Bisphenol A-induced auditory damage and identify potential therapeutic targets, but also establish a theoretical basis for early clinical diagnosis and precision treatment.

## Data Availability

The original contributions presented in this study are included in the article/Supplementary material, further inquiries can be directed to the corresponding authors.

## Footnotes

1
https://pubchem.ncbi.nlm.nih.gov/

2
https://www.bindingdb.org/rwd/bind/index.jsp

3
https://ctdbase.org/

4
http://www.swisstargetprediction.ch/

5
http://targetnet.scbdd.com/

6
https://cn.string-db.org/

7
https://tox.charite.de/protox3/
