# Supplementary material for: Bisphenol A induces ototoxic injury by ferroptosis pathway: integrated analysis of network toxicology, RNA-sequencing and experimental validation
Source: Front Mol Neurosci. 2026 Jul 28;19:1829676. doi: 10.3389/fnmol.2026.1829676 (PMC13457743; doi:10.3389/fnmol.2026.1829676)
Supplement: Supplementary file 1 [file Table_1.docx]

**Supplementary Material**

Supplementary Figure S1. Cell viability of HEI-OC1 cells after Bisphenol A treatment.
